# Anaesthetic Management for Mastectomy in a Male With Unilateral Gynecomastia: The Utilization of Thoracic Segmental Spinal Anaesthesia and Erector Spinae Plane Block

**DOI:** 10.7759/cureus.47502

**Published:** 2023-10-23

**Authors:** Amreesh Paul, Anjali Borkar

**Affiliations:** 1 Anaesthesiology, Jawaharlal Nehru Medical College, Datta Meghe Institute of Higher Education and Research, Wardha, IND

**Keywords:** ultrasound-guided erector spinae plane block (espb), ultrasound guided espb, espb, gynecomastia, breast analgesia, erector spinae plane block (espb), thoracic segmental spinal anesthesia

## Abstract

Males are frequently affected by gynecomastia, a benign proliferative glandular tissue condition of the breast. Gynecomastia is usually treated with surgery to remove breast tissue. Using erector spinae plane block and thoracic segmental spinal anaesthesia in place of typical general anaesthesia during breast procedures has become more common in recent years. This case report presents the management of a 24-year-old male with long-standing left breast gynecomastia. Using a combination of erector spinae plane block and thoracic segmental spinal anaesthesia, the patient had the breast tissue excised. The regulation of the neuroendocrine stress response, lower need for analgesics after surgery, and decreased postoperative nausea and vomiting are among the many benefits of the anaesthetic methods. With better patient outcomes, fewer surgical complications, and efficient postoperative pain management, these methods offer a compelling substitute for general anaesthesia. The range of surgical scenarios in which these techniques can be applied could be expanded by additional research and clinical experience.

## Introduction

The male breast is relatively free from pathologic involvement. Two main pathologies that occur are gynecomastia and carcinoma of the male breast. Gynecomastia is the most common benign proliferative glandular tissue disorder of the male breast [1,2]. It accounts for about 60% of the pathologies of the male breast. It can be bilateral or unilateral [3]. It is mainly seen in the neonatal period, during puberty, and in elderly males above the age of 50 [4]. Gynecomastia is predominantly bilateral and physiological. At the time of puberty, the level of androgens in males is high. The conversion of these androgens to estrogens may lead to gynecomastia [5]. Suppression of androgens or an increase in estrogens can cause estrogen to overcome the inhibitory action of androgens to stimulate breast tissue proliferation [6]. Treatment involves surgical resection of the breast tissue.

Due to its capacity to reduce the dangers, difficulties, and adverse effects of general anaesthesia, thoracic segmental spinal anaesthesia has been increasingly popular in recent years. Anesthesiologists are increasingly choosing to use subarachnoid blocks in the mid-thoracic level for breast procedures due to their specific anatomical advantages, including the widest dural-spinal cord distance [7]. In addition to maintaining stable intraoperative hemodynamics and significantly lowering the incidence of postoperative nausea and vomiting as well as other side effects frequently linked to general anaesthesia, this method offers a potent combination of motor and sensory block. Concurrently, the usage of erector spinae plane block has become a viable method for providing analgesia following breast procedures. This technique blocks sensation in several areas of the chest wall by applying a local anaesthetic next to the vertebral process, which is placed well away from the pleura, nerves, and main blood arteries. Because of this, erector spinae plane blocks are now known to be a safer option than more conventional ones like epidural or para-vertebral blocks. It has been shown in numerous case studies to be effective in providing significant relief from visceral as well as somatic pain [8].

This case presentation provides a compelling example of the potential benefits of these novel anaesthetic techniques in improving patient care and reducing the drawbacks of conventional anaesthetic methods. It documents the effective implementation of these techniques in a 24-year-old male patient with a long-standing left breast enlargement.

## Case presentation

**Figure 1:**
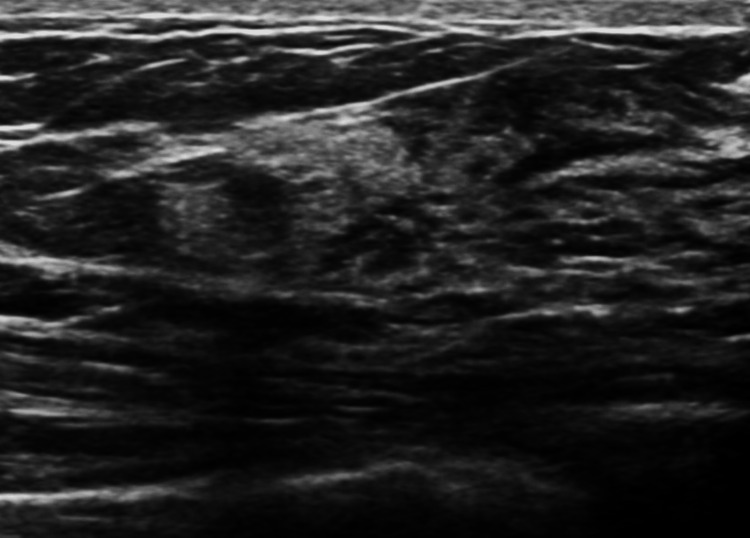
Ultrasound of the lump

A 24-year-old male came to the hospital complaining of a lump in the left side of his chest for nine years. The lump was insidious in onset and gradually progressive in size. Initially, it was the size of 0.5 cm, which progressed to the current size of 10 × 8 × 4 cm. There is no history of trauma or nipple discharge. Ultrasound of the breast revealed diffuse glandular type of gynecomastia (Figure 1).

Preoperative evaluation revealed no underlying medical conditions, an adequate level of physical stamina, and a history of tobacco smoking for the previous three years. The patient's vital signs, included a 74 beats per minute pulse, a 110/80 mmHg blood pressure, 16 breaths per minute respiration, and 99% oxygen saturation on room air. In addition, the patient had a clear airway as evidenced by their satisfactory mouth opening and Mallampati class II classification. Bilateral lung auscultation indicated equal and clear air entry and a thorough systemic evaluation returned normal results. The results of the ECG, radiographic evaluations, and laboratory testing were all within normal limits for a healthy individual. It was decided to employ thoracic segmental spinal anaesthesia as the principal technique, along with the utilisation of erector spinae plane block for pain management following surgery, after conferring with the surgical team and having informed discussions with the patient. Prior to the surgery, the patient was fully informed about the planned anaesthetic procedure, its risks and benefits, and his signed consent was obtained.

Anaesthetic management

A pre-medication dose of 0.5 mg alprazolam tablet was given to the patient the evening before the surgery, and he was kept fasting for eight hours. The patient was moved to the operating room on the day of the procedure, where multipara monitors were applied in compliance with ASA monitoring guidelines. After recording the patient's baseline vital signs, an 18G intravenous cannula was placed in the patient's right arm. Intravenous fluids and drugs including ceftriaxone (1 g i/v), midazolam (2 mg i/v), and ondansetron (4 mg i/v) were given. The anaesthetic procedure was thoroughly explained again to the patient.

**Figure 2:**
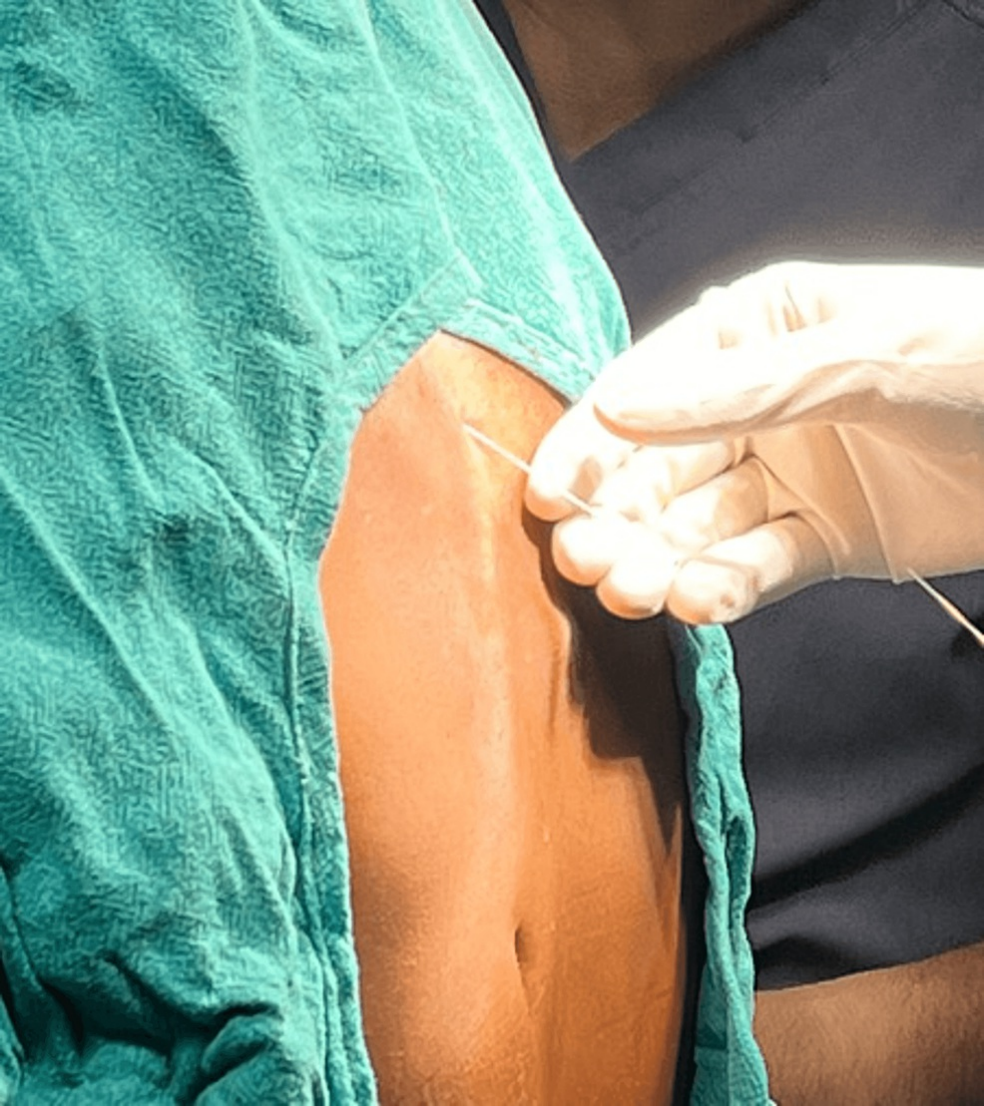
Location of 27G needle placement for erector spinae plane block

Following strict aseptic measures, and with the patient in a seated position, a local anaesthetic skin wheal was created at the level of the left T4 vertebra using a 27G needle. According to Luftig et al. [9], the erector spinae plane at the left T4 transverse process level was located using ultrasonic guidance and a 3.0-13.0 MHz frequency probe. After aspiration for blood was confirmed to be negative, the erector spinae plane block was performed with a 23G 90 mm spinal needle, administering 60 mg of 0.25% levobupivacaine (Figure 2).

**Figure 3:**
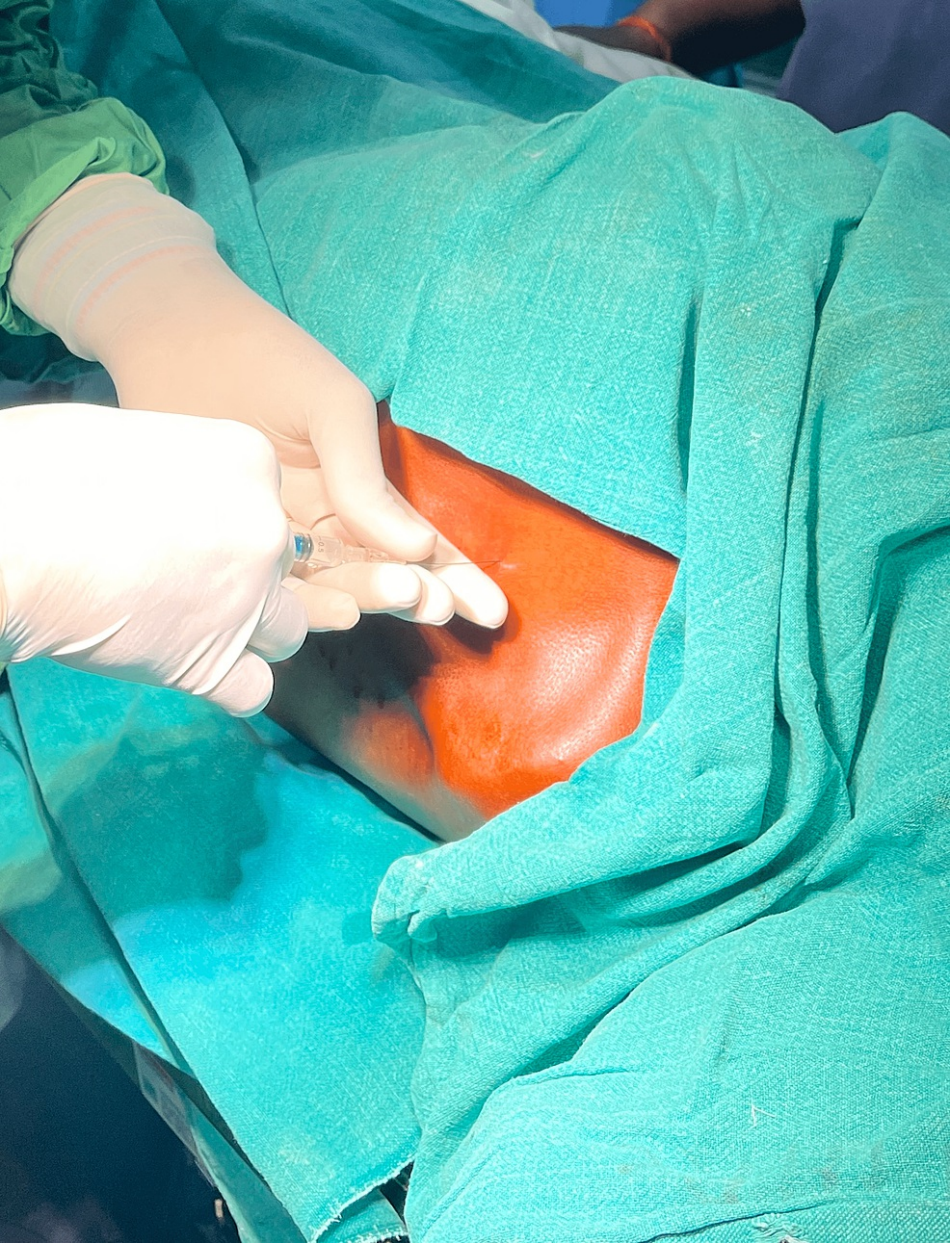
Administration of thoracic segmental spinal anaesthesia

With the patient in the lateral position, administration of thoracic segmental spinal anaesthesia was done at T4-T5 interspace using a landmark-guided technique (from the angulus inferior scapulae) with a 27G 90 mm Quincke needle. Injection levobupivacaine isobaric (0.5%) 6mg and injection fentanyl 25 mcg (intrathecal) were injected after the free flow of cerebrospinal fluid was confirmed (Figure 3). Isobaric levobupivacaine was used for better hemodynamic stability, as suggested by Solakovic [10]. After spinal anaesthesia was administered, the patient was placed supine and the spinal onset of sensory block was monitored at 5, 10, and 15 minutes after the block was administered. The pin-prick method was used to test for loss of sensation in the mid-axillary line from C8 to T7. Using a nasal cannula, the patient received oxygen at a rate of two litres per minute. Following confirmation of adequate sensory block, the procedure was started.

Course of surgery

Verbal communication was maintained with the patient throughout the procedure. Under strict aseptic precautions, the parts were painted and draped. A circumareolar incision was taken on the left breast. The incision was deepened, and the nipple-areolar complex was lifted from the underlying tissue. Five mm of tissue was kept in situ to preserve its blood supply. The surrounding skin was dissected circumferentially from the fat tissue until the pectoral fascia was reached. The fat was lifted from its base from the underlying pectoral muscle. Hemostasis was achieved, and a negative suction drain was placed. The wound was closed in layers, and the dressing was done.

The surgery was conducted for ninety minutes, and intra-operative sedation was not required during this period. No evidence of surgical pain, respiratory depression, and loss of motor sensation in the upper and lower limbs was observed. The patient’s hemodynamics were monitored throughout the procedure and were found stable. After the surgery, the level of sensory block was assessed, and it remained the same. The patient was then shifted to the intensive care unit for further monitoring.

Postoperative monitoring and discharge

The level of sensory block weaned off after 4 hours from the time of administration of the block. Visual analogue scores for pain assessment of the patient at 0, 1, 2, 4, 8, 16, and 24 hours were 0, 0, 1, 2, 5, 5, 4, and 3, respectively. Post resolution of the block, injection of paracetamol 1 g i/v was administered to the patient sixth hourly for pain relief. He was shifted to the surgical ward after 24 hours. The patient was closely monitored during the entire stay of hospitalization. On postoperative day 5, he was discharged from the hospital.

## Discussion

When compared to general anaesthesia, neuraxial blockade has many advantages. These benefits include the inhibition of the neuroendocrine stress response, a decreased risk of postoperative nausea and vomiting, and a decreased requirement for analgesics. Compared to other spinal segments, the thoracic level is distinguished by a smaller volume of cerebrospinal fluid and thinner thoracic nerve roots. As a result, only a small amount of anaesthetic drugs are required to accomplish very strong and effective blockades in these regions [11]. As revealed by recent MRI investigations, the intrathecal space is significantly wider in the mid-thoracic to lower-thoracic area [7]. With respect to dermatomal levels, this provides an additional safety margin for the administration of thoracic spinal anaesthesia due to the lower amount of anaesthetic agent required and its efficient distribution and fixation. It is imperative to acknowledge a critical issue pertaining to thoracic spinal anaesthesia, which is the possible effect on ventilation in situations where a greater block level is attained. However, the diaphragm is uninvolved because the C3, C4, and C5 nerve roots supply it with innervation [7]. The lowest block level in this case scenario was T7, which suggests no motor blockade in the lower limbs, improving patient satisfaction. With a significantly lower incidence of post-operative nausea and vomiting than general anaesthesia, thoracic segmental spinal anaesthesia has several advantages over it, including improved patient comfort, hemodynamic stability, decreased opioid use, early mobilisation, and efficient pain management [12]. However, it is essential to be aware of the possible side effects of segmental spinal anaesthesia. These include but are not limited to hypotension, bradycardia, transient hearing loss, retention of urine, needle trauma to the spinal cord, meningitis, vertebral canal hematoma, spinal cord ischemia, total spinal anaesthesia, and cardiovascular collapse [11].

Significant postoperative pain following breast surgery is common, which might impede the patient's quick recovery. It is difficult to manage postoperative pain effectively because of the complex innervation of the breast. Although a number of regional anaesthetic methods have been developed to treat this pain, each has its own setbacks. Comparable to the notable profiles of retrolaminar and paravertebral blocks, the erector spinae plane block offers a safe and efficient way to deal with this problem [13]. The erector spinae plane block, when given at the T4 level with 25ml of local anaesthetic, has shown exceptional postoperative analgesic efficacy [14]. Reduced pain scores, less dependency on opioids, and a lower rate of postoperative nausea and vomiting are some of its benefits [15]. The complications associated with this block include local anaesthetic systemic toxicity, vascular or pleural puncture, and pneumothorax [16].

This case study describes the use of erector spinae plane block and thoracic segmental spinal anaesthesia in a male patient, aged 24 years, who had a nine-year history of left breast enlargement as a result of gynecomastia. These anaesthetic methods introduce a number of benefits that should be discussed and represent an evolution from traditional general anaesthesia. In this instance, the erector spinae plane block in conjunction with thoracic segmental spinal anaesthesia showed impressive clinical results. Throughout the procedure, the patient's vital signs remained stable, no intraoperative anaesthesia was required, and the patient felt very little pain. These anaesthetic methods aided in a speedy recovery after surgery while also lowering the neuroendocrine stress response.

## Conclusions

We would like to suggest that a feasible anaesthetic strategy for breast procedures is the combination of erector spinae block with thoracic segmental spinal anaesthesia, based on our practical observations. As it has several benefits over general anaesthesia, thoracic segmental spinal anaesthesia is becoming more and more popular for a variety of abdominal and thoracic surgical procedures. Concurrently, the erector spinae plane block shows exceptional effectiveness in relieving postoperative pain after breast procedures. When combined, these two methods have many advantages, such as improved intra-operative hemodynamic stability, increased patient compliance, and efficient post-operative pain control, which is particularly beneficial for a certain group of patients undergoing breast surgery. With each patient being distinctive and each surgical procedure having multiple modalities of anaesthesia, the anaesthesiologists must expand their horizons to meet the patient’s best interest. This case study illustrates the efficacy and safety of these anaesthetic techniques, demonstrating how they may enhance patient outcomes and satisfaction. These methods offer useful substitutes for conventional general anaesthesia as the science of anesthesiology develops, particularly for breast procedures. These techniques may continue to find new uses and advantages in a range of surgical scenarios with additional research and clinical practice.
